# A Mobile Health Intervention for HIV Prevention Among Racially and Ethnically Diverse Young Men: Usability Evaluation

**DOI:** 10.2196/11450

**Published:** 2018-09-07

**Authors:** Hwayoung Cho, Dakota Powell, Adrienne Pichon, Jennie Thai, Josh Bruce, Lisa M Kuhns, Robert Garofalo, Rebecca Schnall

**Affiliations:** 1 School of Nursing Columbia University New York, NY United States; 2 Division of Adolescent Medicine Ann & Robert H Lurie Children's Hospital of Chicago Chicago, IL United States; 3 Birmingham AIDS Outreach Birmingham, AL United States; 4 Department of Pediatrics, Feinberg School of Medicine Northwestern University Chicago, IL United States

**Keywords:** mobile apps, mobile health, information technology, health information technology, usability evaluation, adolescents, HIV prevention, men who have sex with men

## Abstract

**Background:**

Mobile health (mHealth) apps have the potential to be a useful mode of delivering HIV prevention information, particularly for young men (13-24 years) who account for 21% of new HIV diagnoses in the United States. We translated an existing evidence-based, face-to-face HIV prevention curriculum into a portable platform and developed a mobile Web app: MyPEEPS Mobile.

**Objective:**

The purpose of this study was to assess the usability of MyPEEPS Mobile from both expert and end user perspectives.

**Methods:**

We conducted a heuristic evaluation with five experts in informatics to identify violations of usability principles and end user usability testing with 20 young men aged 15 to 18 years in New York, NY, Birmingham, AL, and Chicago, IL to identify potential obstacles to their use of the app.

**Results:**

Mean scores of the overall severity of the identified heuristic violations rated by experts ranged from 0.4 and 2.6 (0=no usability problem to 4=usability catastrophe). Overall, our end users successfully completed the tasks associated with use case scenarios and provided comments/recommendations on improving usability of MyPEEPS Mobile. The mean of the overall Post-Study System Usability Questionnaire scores rated by the end users was 1.63 (SD 0.65), reflecting strong user acceptance of the app.

**Conclusions:**

The comments made by experts and end users will be used to refine MyPEEPS Mobile prior to a pilot study assessing the acceptability of the app across diverse sexual minority young men in their everyday lives.

## Introduction

### Background

With the rapid proliferation of mobile phone ownership across the world, use of mobile technologies in health care has expanded [1]. More than 325,000 mobile health (mHealth) apps were available globally on Apple iTunes and Google Play in 2017, and the number of mHealth apps continues to increase [2]. The ubiquitous nature of mobile phones brings convenience to everyday lives and creates opportunities to deliver health interventions in a portable format with enhanced privacy, increasing accessibility to the health interventions particularly tailored for stigmatized and disenfranchised populations [3-5].

**Figure 1:**
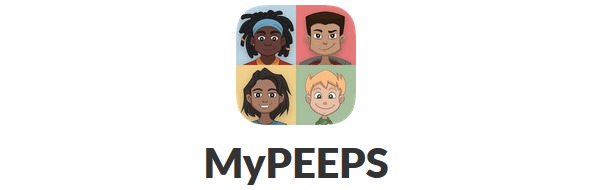
Four YMSM (young men who have sex with men) avatars on the MyPEEPS Mobile app.

mHealth technology specifically has the potential to be a useful delivery mode of health information because it allows for the dissemination of information quickly and broadly [6-8].

For the success of health information technologies, usability must be considered from the start of system development [9], yet few mHealth apps have undergone rigorous usability evaluation prior to their dissemination [10]. Usability factors remain one of the major obstacles to adoption of mHealth technologies because mHealth apps produced with poor quality are difficult to use, or are misused, which can lead to unintended consequences [11-13]. Therefore, usability evaluations are necessary to identify usability violations, guide system modification, and enhance technology acceptance by end users [14]. To provide the most effective and thorough usability evaluation results, a combination of usability evaluation techniques, including both experts and intended end users, during the evaluations is recommended [15,16].

### Study Context: MyPEEPS Intervention

In 2016, of the 39,782 people in the United States newly infected with HIV, 21% were youth ages 13 to 24 years and 81% of incident cases among these youth were diagnosed in young men who have sex with men (YMSM), disproportionately occurring in African-American/black and Latino/Hispanic men [17]. An original Male Youth Pursuing Education, Empowerment & Prevention around Sexuality (MyPEEPS) intervention is a theory-driven (ie, social cognitive theory) [18], manualized HIV prevention curriculum developed for racially and ethnically diverse YMSM to address the need for an evidence-based HIV prevention intervention for this population [19]. The group-based, in-person intervention was found to be efficacious on reducing sexual risk, specifically sexual risk while under the influence of alcohol or drugs, in a 12-week feasibility trial. Nonetheless, participant engagement proved challenging due to travel distance and logistics around scheduling a group-based intervention.

The use of mobile apps has been a popular way, particularly for YMSM, to get health information, connect with gay friends, and seek sex partners [20]. With the great promise of mHealth technology, we translated the existing face-to-face intervention into a mobile platform using an iterative design process [21,22]. The mobile Web app, MyPEEPS Mobile, was implemented by software developers at Little Green Software. MyPEEPS Mobile is guided by four YMSM avatars (ie, Philip aka P, Artemio, Nico, and Tommy; Figure 1) who manage their sexual health against a backdrop of personal, family-based, and relational challenges, and deliver the HIV prevention information to the end users. MyPEEPS Mobile consists of 21 activities divided into four modules or “PEEPScapades.” The activities include didactic content, graphical reports, videos, and true/false and multiple-choice quizzes. A user is required to complete the activities in consecutive order. On completing each activity, the user receives a trophy as a reward to promote continued participation. The purpose of this study was to assess the usability of the mHealth intervention, MyPEEPS Mobile, from the perspectives of experts and end users.

## Methods

### Overview

We conducted two types of rigorous usability evaluations of MyPEEPS Mobile. First, we conducted a heuristic evaluation with informatics experts to identify violations of usability principles. Next, end user usability testing was conducted with target users, young men who are attracted to other men, to identify obstacles to their use of MyPEEPS Mobile. The Institutional Review Board (IRB) of Columbia University Medical Center in New York, NY, served as the central IRB for this study and approved all study activities.

### Heuristic Evaluation

#### Sample Selection

Five informaticians were invited via email to participate in a heuristic evaluation of MyPEEPS Mobile. The sample size was chosen in accordance with Nielsen’s recommendation to include three to five heuristic evaluators, as no additional information is likely to be produced with a larger sample [23]. Qualifications of the experts included (1) at least a Master’s degree in the field of informatics and (2) training in human-computer interaction. These qualifications were essential since the quality of the heuristic evaluation is dependent on the skills and experience of the usability experts [24].

#### Procedures

Heuristic evaluators were given a description of the full functionality of MyPEEPS Mobile. Each heuristic evaluator completed each of the 21 activities within the app (Textbox 1) at least once.

**Summary of the 21 activities on MyPEEPS Mobile.**

**I. Intro**

1. Welcome to MyPEEPS

- Introduction to the app explaining what the user is to expect. User inputs name, telephone number, email address, and how they prefer to get notifications.

2. BottomLine

- User is asked the farthest they will go with a one-time hookup in a number of sexual scenarios (when I give head, when I top, etc) and given a selection of responses about what they will and won’t do and how they will do it (always use a condom, won’t use a condom, will never do this).

3. Underwear Personality Quiz

- User completes a personality quiz and is introduced to the avatars that they will be seeing in the app. Avatars’ personality traits and identities are shared with “gossip.”

4. My Bulls-I

- User is asked to think about their important identity traits and create a list of their top five favorite or best identity traits after seeing an example of the activity done by one of the app avatars, P.

**II. #realtalk**

5. P’s On-Again Off-Again BottomLine

- Video of a text conversation between two avatars, P and Nico, about P’s new relationship and P ignoring his BottomLine. The user is asked to complete questions about why P should be concerned about his BottomLine with a new partner. There are two videos with two sets of questions (video → questions → video → questions).

6. Sexy Settings

- User is presented with a setting in which sex could be taking place and is given one potential threat to a BottomLine and are asked to select another potential threat for the given setting.

7. Goin’ Downhill Fast

- User is presented with information about drugs and alcohol and how they can affect a BottomLine. Resources for additional information about drugs and alcohol are provided. After reading through the information, users complete a set of questions about the potential impact of drugs and alcohol on their BottomLine.

8. Step Up, Step Back

- User is introduced to identity traits that may identify them as a VIP (privileged)/non-VIP (nonprivileged) and then asked a series of identity-related questions. An avatar representing the user moves back and forth in a line for a night club, relative to the avatars in the app, as questions are answered.

9. HIV True/False

- User completes a series of true/false questions related to HIV, with information following a correct answer.

10. Checking in on Your BottomLine

- User is given the opportunity to review and make changes to their BottomLine, taking into consideration any information that they may have learned from completing the activities prior to this check-in.

**III. Woke Up Like This**

11. P Gets Woke About Safer Sex

- User is presented a scenario about P trying to make his way to the clinic to get tested. P experiences difficulties and rude behavior, and the user is presented with recommendations for managing anger and frustration.

12. Testing With Tommy

- User watches a video about a character’s (Tommy) experience with getting tested for HIV for the first time. The video presents a clinic scenario and a discussion with the HIV testing and prevention counselor. Information about accessing HIV testing services is provided.

13. Well Hung

- User is introduced to the association of HIV transmission risk with different sexual behaviors categorized into no risk, low, medium, and high risk. The user completes an activity dragging and dropping a given sexual activity onto the risk category associated with the sex act.

14. Ordering Steps to Effective Condom Use

- User is presented with 12 steps for effective condom use and must correctly order the steps by selecting them chronologically from a list of all the steps.

15. Checking in on Your BottomLine Again

- User is again given the opportunity to review and make changes to their BottomLine, taking into consideration any information that they may have learned from completing the activities prior to this check-in.

**IV. Making Tough Situations LITuations**

16. Peep in Love

- User is presented a scene where P is with his partner and wanting to engage in sexual activity without protection, a violation of P’s BottomLine. The user is then asked about possible feelings and emotions that P might be having in the scene. An overview of the feelings is given at the end so the user can see the possible “swirl of emotions” from the scenario. User is then given information about how to communicate effectively with sex partners so that they can maintain their BottomLine.

17. 4 Ways to Manage Stigma

- User is presented with four stigma management strategies, then a scene for each of the four app avatars and asked to answer which strategy each character is using in the scene.

18. Rubber Mishap

- User is asked to complete a series of questions relating to condom usage as the screen shakes to mimic being under the influence of drugs or alcohol.

19. Get a Clue!

- Jumbled scenarios are created using either a shake of the phone or press of a button. User answers from given options how they would act in the scenario, keeping the BottomLine and communication strategies in mind.

20. Last Time Checking in on Your BottomLine

- User is again given the opportunity to review and make changes to their BottomLine, taking into consideration any information that they may have learned from completing the activities prior to this check-in.

21. BottomLine Overview

- User is presented with a list of their BottomLine selections since the initial activity and subsequent check-ins.

Experts were instructed to think-aloud as they evaluated the app. The process was recorded using a TechSmith Morae Recorder [25], which enables the researcher to record and analyze the audio recording and screenshots captured during the heuristic evaluation. Following completion of the tasks, heuristic evaluators were asked to rate the severity of the violations using an online version of the Heuristic Evaluation Checklist developed by Bright et al [26], based on Nielsen’s 10 heuristics [27]. Each heuristic was evaluated by one or more items and the overall severity of the identified heuristic violations were rated into five categories: no problem (0), cosmetic problem only (1), minor problem (2), major problem (3), and usability catastrophe (4). The evaluators were also asked to provide additional comments regarding the user interface. After the surveys were completed, evaluators received US $150 as compensation for their time.

#### Data Analysis

All experts’ comments about usability problems on the evaluation form and from the Morae recordings were compiled and reviewed by two research team members. Discrepancies in coding the data according to the usability factors of Nielsen’s 10 heuristics were discussed until consensus was achieved. Mean severity scores were calculated for each heuristic principle.

### End User Usability Testing

#### Sample Selection

For end user usability testing, potential participants were recruited from local community organizations through the use of passive and active methods (ie, convenience sampling; flyers, posting on social media, and direct outreach at community-based organizations) in New York, NY; Birmingham, AL; and Chicago, IL. Eligibility criteria were (1) between 13 and 18 years of age, (2) self-identified as male, (3) male sex assigned at birth, (4) understand and read English, (5) living within the metropolitan area of one of the three cities, (6) ownership of a mobile phone, (7) sexual interest in men and having either kissed another man or plans on having sex with a man in the next year, and (8) self-reported HIV-negative or unknown status. A sample of 20 participants was anticipated to be sufficient because prior research suggests an increasing benefit with samples up to 20 in usability testing (ie, the minimum percentage of problems identified rose from 82% up to 95% when the number of users was increased from 10 to 20) [28].

#### Procedures

All participants were given a brief explanation of MyPEEPS Mobile. The first 10 participants were provided with use case scenario version 1 (Textbox 2); once data saturation was achieved, the remaining 10 participants were provided with use case scenario version 2 (Textbox 3). Participants were asked to complete tasks using MyPEEPS Mobile on an iOS simulator for Windows computers. While participants were doing the tasks, the computer screen was video recorded using iMotions software (iMotions Biometric Research Platform 6.0, iMotions A/S, Copenhagen), which enables researchers to present images or screen/scene recordings and synchronize data from a variety of hardware platforms, if needed (eg, eye-tracking data), simultaneously. After the participants completed the tasks, they were then asked to watch a recording of their task performance on the computer screen. Participants were encouraged to retrospectively think-aloud and asked to verbalize their thoughts about the tasks they completed while watching a replay of the screen recordings. The process, including participants’ verbal comments, was audio recorded using Morae [25]. As part of the usability assessment, participants were asked to rate the app’s usability using the third version of the Post-Study System Usability Questionnaire (PSSUQ) [29] administered via Qualtrics (Provo, UT, USA) following the testing of MyPEEPS Mobile. The third version of the PSSUQ is a 16-item survey instrument to assess system usability on a scale ranging from 1 (strongly agree) to 7 (strongly disagree) including a neutral midpoint. A lower score on the PSSUQ indicates higher perceived usability of the app. The study visit took approximately 2 hours and participants were compensated US $40 to US $50 for their time. Interested individuals were consented for participation with a waiver of parental permission for minors.

**Use case scenario version 1 (N=10).**

1. Log in to the MyPEEPS Mobile

- Click on activity #1 “Welcome to MyPEEPS!” to begin
- Collect the trophy from activity #2, “BottomLine”
- Collect the trophy from #3, “Underwear Personality Quiz”
- Collect the trophy from #4, “My Bulls-I”
- Collect the trophy from #5, “P’s On-Again Off-Again BottomLine”
- Collect the trophy from #7, “Goin’ Downhill Fast”
- Collect the trophy from #8, “Step Up, Step Back”
- Collect the trophy from #9, “HIV True/False”
- Collect the trophy from #10, “Checking in on Your BottomLine”
- Collect the trophy from #13, “Well Hung??”
- Collect the trophy from #18, “Rubber Mishap”

2. View Settings

3. Log Out

**Use case scenario version 2 (N=10).**

1. Log in to the MyPEEPS Mobile

- Click on activity #1 “Welcome to MyPEEPS!” to begin
- Collect the trophy from activity #2, “BottomLine”
- Collect the trophy from #6, “Sexy Settings”
- Collect the trophy from #10, “Checking in on Your BottomLine”
- Collect the trophy from #13, “Well Hung??”
- Collect the trophy from #15, Checking in on Your BottomLine Again”
- Collect the trophy from #17, “4 Ways to Manage Stigma”
- Collect the trophy from #19, “Get a Clue!”
- Collect the trophy from #20, “Last Time Checking in on Your BottomLine”
- Collect the trophy from #21, “BottomLine Overview”

2. View Settings

3. Log Out

#### Data Analysis

Data analysis was based on the audio/video recordings collected by Morae [25] and iMotions software. Participants’ verbalizations from the audio recordings were transcribed verbatim. Notes of critical incidents, characterized by comments, silence, repetitive actions, and error messages, were compiled from the recordings. Content analysis, a technique for making replicative and valid inferences from data, was performed by two research team members by reviewing the transcripts and critical incidents to identify common usability concerns. A third reviewer consulted in instances of uncertainty or discrepancy in the content analysis. Results from the PSSUQ were analyzed using Stata SE 14 (StataCorp LP, College Station, TX, USA) to calculate the descriptive statistics to complement the findings from the usability assessment.

## Results

### Heuristic Evaluation

The mean age of the heuristic evaluators was 46.2 (SD 8.9) years, and mean years of experience in informatics that they had was 13.0 (SD 4.5) years. All the heuristic evaluators were female, 60% (n=3) were Asian, and 40% (n=2) were white. Mean scores and sample comments from the heuristic evaluation were organized into Nielsen’s 10 usability heuristics (Table 1) [27,30]. The mean scores of the overall severity of the identified heuristic violations ranged from 0.4 and 2.6, in which scores closest to 0 indicate a more usable app.

**Figure 2:**
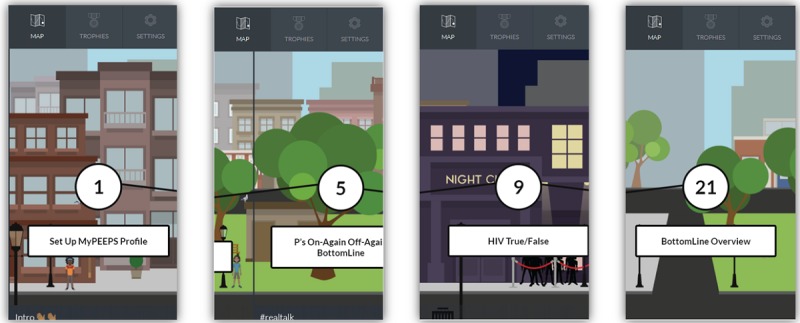
Map on MyPEEPS Mobile.

**Figure 3:**
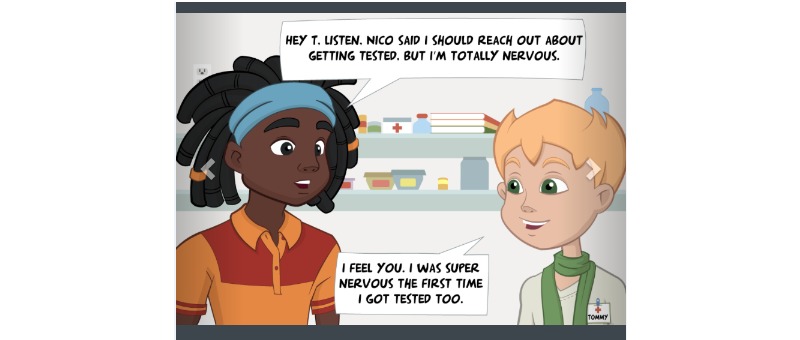
Comics with unclear navigation.

The heuristic principle identified as the most in need of refinement was “user control and freedom” (mean 2.60, SD 1.14). Experts pointed out that MyPEEPS Mobile did not allow users the ability to move forward and backward: the “Back to Map” button only appears at the beginning of each activity. The second heuristic most identified for improvement was visibility of system status (mean 2.20, SD 0.45). A total of 21 activities divided into four PEEPScapades (Textbox 1) were displayed along a virtual “map” within MyPEEPS Mobile (Figure 2). The heuristic evaluators indicated that it was unclear which PEEPscapade they were in on the map, and the app should keep users informed about what was going on. Moreover, heuristic evaluators identified that the white navigation arrows used to advance through the “Testing with Tommy” activity and related comics (eg, illustrating what symptoms to look for when it comes to getting tested and treated for sexually transmitted diseases) were not clearly visible (Figure 3).

**Figure 4:**
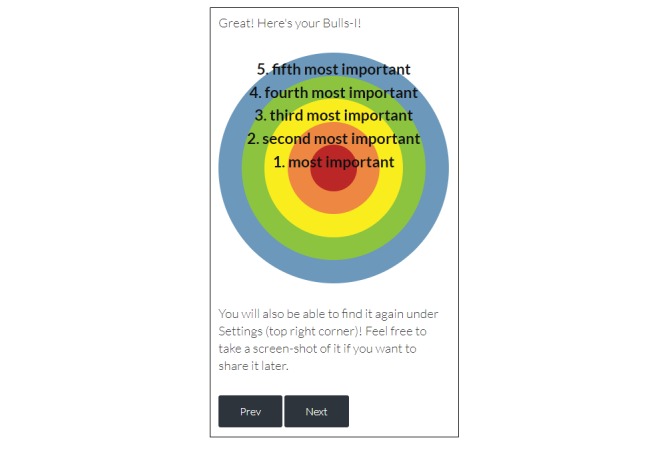
My Bulls-I activity.

**Figure 5:**
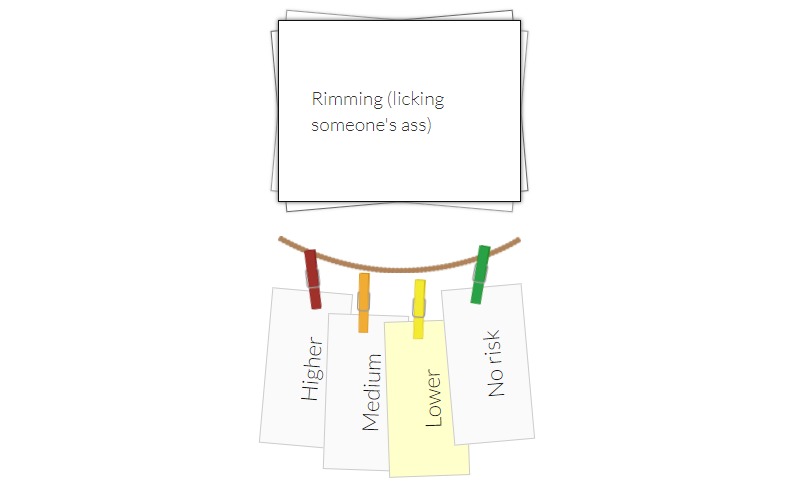
Well Hung activity.

In response to the usability factor “help and documentation” (mean 1.60, SD 0.89), one expert pointed to the lack of an instruction manual on how to navigate the app as a major concern. To improve “match between system and the real world” (mean 1.40, SD 0.89), experts recommended that an individual’s five important identity traits in the “My Bulls-I” activity (ie, move most important at the top followed by second through fifth; Figure 4) and response options for risk level in the “Well Hung” activity (ie, move no risk to far left side, followed by greater levels of risk to the right; Figure 5) be listed in a natural/logical order.

### End User Usability Testing

The mean age of the end users was 17.4 (SD 0.88, range 15-18) years. Demographic characteristics including race, ethnicity, current student status, and education level are reported in Table 2. Sexual orientation was characterized using a gradient scale ranging from exclusively gay/homosexual to exclusively heterosexual, to capture the fluidity of sexuality at the time of the survey. Descriptive statistics on technology use, including type of mobile phone, social media sites, and apps are reported in Table 3. The majority of participants (85%, n=17) reported almost constant internet use (more than several times a day). The same percentage (85%, n=17) of participants reported using mobile devices (eg, mobile phone, tablet, and cell phone) as opposed to using laptop/desktop (15%, n=3) to access the internet in the past month. The mean duration of participants’ use of mobile apps on a mobile phone per day was 9.40 (SD 5.52) hours.

**Table 1:** Mean severity scores^a^ and sample comments from the heuristic evaluation.

Nielsen’s 10 usability heuristics	Mean (SD)	Sample comments
Visibility of system status	2.20 (0.45)	Unclear where I am on the map
Match between system and the real world	1.40 (0.89)	Five identity traits should be listed from top-most important to bottom-least important
User control and freedom	2.60 (1.14)	Unavailable “Back to Map” (only available at the beginning of each activity)
Consistency and standards	0.40 (0.89)	Hints should be consistently provided for both incorrect/correct answers
Help users recognize, diagnose, and recover from errors	1.00 (1.00)	Error messages should provide with additional information of incorrect answers
Error prevention	1.00 (1.41)	Data entry boxes should contain default values (eg, email address/phone number)
Recognition rather than recall	1.00 (1.00)	Need instructions on how to answer; vertical compression to see buttons
Flexibility and efficiency of use	0.80 (1.30)	Have an option of directly texting a link to friends
Esthetic and minimalist design	0.80 (0.84)	Visual layout of “BottomLine Overview” should be redesigned for simplicity
Help and documentation	1.60 (0.89)	No manual on how to navigate the app

^a^Rating score from 0=best to 5=worst; no usability problem (0), cosmetic problem only (1), minor usability problem (2), major usability problem (3), and usability catastrophe (4).

### Positive Comments

Overall, our end users successfully completed the tasks associated with the use case scenarios (Textboxes 2 and 3) and provided positive comments regarding the use of MyPEEPS Mobile. For example, participants liked the design and layout of MyPEEPS Mobile. One participant stated:

The basic structure is that there’s pretty much an outline and you have four boys/men who are kind of the characters that kind of take you along this journey to HIV prevention and sexual health for MSM. This is sort of like fun. I like cartoons, videos, and quizzes.

Also, participants liked the ease of the overall app use. One participant stated:

I think it was pretty easy once I got the hang of moving to the side. I would just click the number and then I would start the quiz. It was simple. It was pretty quick.

### Recommendations

#### General App Use

Participants provided recommendations to improve usability by expressing their frustrations in general use of the app. For example, several participants commented that receiving error messages over and over frustrated them. They suggested that error messages may be more helpful if an explanation is provided for wrong answers, and they preferred to be provided with a correct answer after two wrong attempts. Moreover, participants identified several terms might be difficult to understand for younger participants (eg, ages 13-15 years). They suggested that explanations be provided for potentially unfamiliar terms (eg, related to sex work, such as “client/tricks”). In addition to the supplementary explanation, participants recommended that the key sexual health terms/keywords (eg, give head/get head/top/bottom) be bolded for emphasis. Several participants reported an issue with the “Previous” button at the end of an activity; instead of taking them to the previous page, it would erroneously take them back to the beginning of the activity.

**Table 2:** Demographic characteristics of participants (N=20).

Characteristic		n (%)	New York (n=9), n (%)	Chicago (n=7), n (%)	Birmingham (n=4), n (%)
**Gender identity**					
	Male	20 (100)	9 (100)	7 (100)	4 (100)
**Sexual orientation**					
	Only gay/homosexual	14 (70)	7 (78)	5 (71)	2 (50)
	Mostly gay/homosexual	2 (10)	2 (22)	0 (0)	0 (0)
	Bisexual	3 (15)	0 (0)	2 (29)	1 (25)
	Something else	1 (5)	0 (0)	0 (0)	1 (25)
**Race**					
	White	9 (45)	2 (22)	4 (57)	3 (75)
	Black or African-American	4 (20)	2 (22)	1 (14)	1 (25)
	Hispanic or Latino/Latinx	4 (20)	2 (22)	2 (29)	0 (0)
	Asian or Asian American	2 (10)	2 (22)	0 (0)	0 (0)
	Multiracial	1 (5)	1 (11)	0 (0)	0 (0)
**Ethnicity**					
	Hispanic	9 (45)	6 (67)	3 (43)	0 (0)
	Non-Hispanic	11 (55)	3 (33)	4 (57)	4 (100)
**Current student status**					
	Currently a student	16 (80)	8 (89)	5 (71)	3 (75)
**Highest level of education completed**					
	Grade 8	2 (10)	2 (22)	0 (0)	0 (0)
	Some high school	7 (35)	3 (33)	3 (43)	1 (25)
	High school diploma (GED^a^)	6 (30)	1 (11)	3 (43)	2 (50)
	Some college	5 (25)	3 (33)	1 (14)	1 (25)

^a^GED: General Equivalency Diploma.

#### Specific Activities Within the App

**Figure 6:**
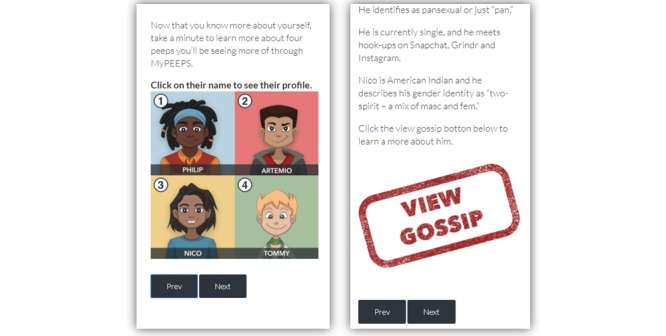
Underwear Personality Quiz.

Participants also provided comments on specific activities within MyPEEPS Mobile. For instance, several participants noted an issue with the features and functions on an activity, Underwear Personality Quiz, in which participants were introduced to four YMSM avatars, and the four avatars’ personality traits were shared with a “gossip” link (Figure 6). Participants had difficulty recalling if they had viewed each avatar’s gossip page. They recommended that an indication mark (eg, checkmark) on the top right corner of each avatar be shown when the avatar’s gossip has been viewed.

A running theme of the app was sexual risk reduction and goal-setting through an activity called the BottomLine. In this activity, participants were challenged to articulate how much risk they were willing to accept for different sexual acts. They were asked to continually reconsider these limits as they progressed through the app (Textbox 1; the activity appears four times throughout the app). At the end, participants were presented with the activity #21, BottomLine Overview, which shows them a chronological overview of how their BottomLine changed as they progressed through the app. The participants were then encouraged to continue to stick to their sexual health goals. Many participants felt that the activity required too much reading (ie, full history of the end user’s BottomLine changes for each sexual act), and/or they did not recognize that the display reflected their own changes (ie, selected responses when they previously completed the four BottomLine activities). One participant commented, “It was like a receipt. It was so long! Oh, I thought it was other people’s [BottomLines].” Rather than a full report, participants suggested that we show only the most current BottomLine responses and highlight where they made changes. One participant stated:

I need only the most current bottom line. I feel like if you click on the bottom line link, you should show like the most current one, and then below that maybe like a link to see the previous ones.

Another participant commented:

Just divide it. Put like all of what I first answered my bottom like, and have it separate in the first square, and then, read it down in a different square, like point out some changes I’ve made to my bottom line since the last time. So, I can compare to the ones on top to see how different they are so they’re not all like mixed.

**Table 3:** Technology use by participants (N=20).

Question		Participants
**Frequency of internet use, n (%)**		
	Almost constantly	17 (85)
	Several times a day	3 (15)
**Devices used in the past month to access the internet, n (%)**		
	Mobile phone/smartphone/mobile handheld device	17 (85)
	Laptop/desktop	3 (15)
**Model/type of mobile phone used, n (%)**		
	iPhone	16 (80)
	Android phone	2 (10)
	Windows phone	1 (5)
	Other (unknown)	1 (5)
**Frequency of using social media sites in the past month, n (%)**		
	Several times a day	17 (85)
	About once a day	2 (10)
	Once every few weeks	1 (5)
**Top sites or apps used for social networking, n (%)**		
	Snapchat	18 (90)
	Instagram	17 (85)
	Facebook	9 (45)
	Twitter	6 (30)
	YouTube	2 (10)
	Tumblr	2 (10)
	LinkedIn	1 (5)
Daily text messages sent and received on cell phones, mean (SD)		183.05 (204.03)
App use on mobile phones (hours/day), mean (SD)		9.40 (5.52)

**Table 4:** Post-Study System Usability Questionnaire (PSSUQ) scores^a^ (N=20).

Construct	Mean (SD)
System quality	1.48 (0.59)
Information quality	1.87 (0.86)
Interface quality	1.55 (0.77)
PSSUQ overall	1.63 (0.65)

^a^Rating score from 1=best to 7=worst (16 items).

Participants’ perceived usability scores rated using PSSUQ [29] are reported in Table 4. Mean of the overall PSSUQ score was 1.63 (SD 0.65), reflecting strong user acceptance of MyPEEPS Mobile.

## Discussion

### Principal Findings

Despite the proliferative use of mobile technologies in health care, few mHealth apps have been released with consideration of their quality through comprehensive and rigorous usability evaluations. Given a lack of evidence-based mHealth apps for HIV risk reduction in high-risk populations, we developed a mobile HIV prevention app for young men ages 13 to 18 years and assessed the app’s usability through a heuristic evaluation with informatics experts and end user testing to identify potential usability issues.

Usability factors remain obstacles to mobile technology adoption. Usability evaluations are foundational to the success of achieving systems that meet human-computer interaction principles and in many cases improve their application in a real-world setting [31]. In the context of rigorous usability testing of the systems, it is critical to choose the most appropriate evaluation methods that meet the study aims and ultimately achieve the goals of the systems. We employed two usability evaluation methods most commonly used in usability studies (ie, a heuristic evaluation and end user testing) to capture different usability perspectives from experts and end users [32]. Similar to prior research, usability experts were more likely to identify usability problems related to general interface features working in a natural and logical order [33,34], whereas end users identified those related to impact on task performance interacting with the app [16]. For example, contrary to feedback received from experts in the heuristic evaluation regarding the usability factor “match between system and the real world,” our end users did not identify the natural/logical order of response options as a problem. Given the natural/logical order matched to the real world, no risk (0) should start on the left ending with higher risk (3) on the right in the Well Hung activity (Figure 5). An example of the attitude of end users regarding the ordering issues was expressed by one participant, who stated, “It was something that I didn’t really pay attention to. I would say like I don’t mind it. It doesn’t matter.” Inclusion of intended end users in the usability testing, in addition to the heuristic experts, enabled us to identify both logic and flow issues as well as functionality most important to end users to support overall engagement with the app [35].

Use case scenarios play an important role in usability evaluations, impacting the quality of usability testing [36]. The use case scenarios should be formulated to facilitate determination of system usability by researchers. Guided by the objectives of usability testing, use case scenarios should include key tasks that can provide valid usability data related to users’ experience with the app use. In this study, we utilized two versions of use case scenarios to capture specific aspects of representative tasks as well as “big picture” issues related to the goals of the app. For example, in the first version of use case scenarios we included tasks that examine end users’ performance on every type of learning activity including comics, animated videos, and games. Given that the running theme throughout the app was sexual risk reduction and goal-setting via the BottomLine activity, in the next version of use case scenarios we included all tasks to test end users’ engagement in BottomLine activities in addition to the learning activities we included in the first version. Using these different versions of robust use case scenarios in end user usability testing enabled us to identify areas where usability was a potential concern and to obtain users’ valuable comments on their overall app use (ie, version 1) as well as specific task performance (ie, version 2), which is a strength of our usability study.

Although findings from our usability evaluations yielded specific feedback regarding ways to improve users’ experience, the overall usability scores rated by the PSSUQ were high, which indicated that our app was perceived as highly usable. The results support the potential for high acceptability of MyPEEPS Mobile. The promising usability of the app provides a foundation for user satisfaction in the planned randomized controlled trial which aims to reduce sexual risk behavior in a high-risk population.

### Limitations

The generalizability of the results may be limited by the study sample, settings, and inclusion/exclusion criteria. Our targeted population was diverse YMSM living within the metropolitan area in New York, Chicago, and Birmingham, and those who had either kissed another man or planned on having sex with a man in the next year. Results may differ in transgender groups, other groups who live in rural areas, or those who have more/less experience in sexual activities. Although the age range for inclusion in this study was between 13 and 18 years, our participants were between 15 and 18 years of age, which may differ in younger adolescent MSM (eg, 13-14 years of age). A limitation of this study was related to the participants’ self-reported data such as end users’ perceived usability scores, which may be influenced and could bias the results.

A limitation of this study was that we conducted the usability evaluations on a computer as opposed to a mobile device. We chose to conduct the evaluations on a computer so that we would be able to analyze the data more effectively using Morae and iMotions. Understanding that there may be differences in computer and mobile device user interactions, we employed an iOS simulator on the computer so that the app can be utilized in the same manner as on a mobile phone. Although still a limitation, the use of the iOS simulator minimized its impact.

### Conclusions

We tested the usability for an evidence-based HIV prevention mobile app intended for diverse YMSM through a heuristic evaluation with informatics experts and end user testing. The use of the two usability assessment methods for a mHealth app added value to this study by producing reliable results of a user interface from experts as well as user interaction with the app from end users. Findings from our rigorous usability evaluations will be used to refine the content, organization, and workflow of MyPEEPS Mobile. Following these refinements, we will conduct a 6-week pilot study to assess end users’ acceptability of the app before beginning a multicity, 12-month efficacy study. Our work highlights the importance of utilizing a rigorous usability approach to refine a mHealth app before it is deployed in a high stakes environment.

## Abbreviations

GED: General Equivalency Diploma
IRB: Institutional Review Board
mHealth: mobile health
MyPEEPS: Male Youth Pursuing Education, Empowerment & Prevention around Sexuality
PSSUQ: Post-Study System Usability Questionnaire
YMSM: young men who have sex with men
